# Simple Scattering: Lipid nanoparticle structural data repository

**DOI:** 10.3389/fmolb.2024.1321364

**Published:** 2024-03-22

**Authors:** Lee Joon Kim, David Shin, Wellington C. Leite, Hugh O’Neill, Oliver Ruebel, Andrew Tritt, Greg L. Hura

**Affiliations:** ^1^ Molecular Biophysics and Integrated Bioimaging Division, Lawrence Berkeley National Laboratory, Berkeley, CA, United States; ^2^ David Shin Consulting, Berkeley, CA, United States; ^3^ Neutron Scattering Division, Oak Ridge National Laboratory, Oak Ridge, TN, United States; ^4^ Scientific Data Division, Lawrence Berkeley National Laboratory, Berkeley, CA, United States; ^5^ Applied Mathematics and Computational Science Division, Lawrence Berkeley National Laboratory, Berkeley, CA, United States; ^6^ Department of Chemistry and Biochemistry, University of California Santa Cruz, Santa Cruz, CA, United States

**Keywords:** lipid nanoparticles, SAS, small-angle scattering, database, vaccines, structure-activity relationship, web application

## Abstract

Lipid nanoparticles (LNPs) are being intensively researched and developed to leverage their ability to safely and effectively deliver therapeutics. To achieve optimal therapeutic delivery, a comprehensive understanding of the relationship between formulation, structure, and efficacy is critical. However, the vast chemical space involved in the production of LNPs and the resulting structural complexity make the structure to function relationship challenging to assess and predict. New components and formulation procedures, which provide new opportunities for the use of LNPs, would be best identified and optimized using high-throughput characterization methods. Recently, a high-throughput workflow, consisting of automated mixing, small-angle X-ray scattering (SAXS), and cellular assays, demonstrated a link between formulation, internal structure, and efficacy for a library of LNPs. As SAXS data can be rapidly collected, the stage is set for the collection of thousands of SAXS profiles from a myriad of LNP formulations. In addition, correlated LNP small-angle neutron scattering (SANS) datasets, where components are systematically deuterated for additional contrast inside, provide complementary structural information. The centralization of SAXS and SANS datasets from LNPs, with appropriate, standardized metadata describing formulation parameters, into a data repository will provide valuable guidance for the formulation of LNPs with desired properties. To this end, we introduce Simple Scattering, an easy-to-use, open data repository for storing and sharing groups of correlated scattering profiles obtained from LNP screening experiments. Here, we discuss the current state of the repository, including limitations and upcoming changes, and our vision towards future usage in developing our collective knowledge base of LNPs.

## 1 Introduction

**FIGURE 1:**
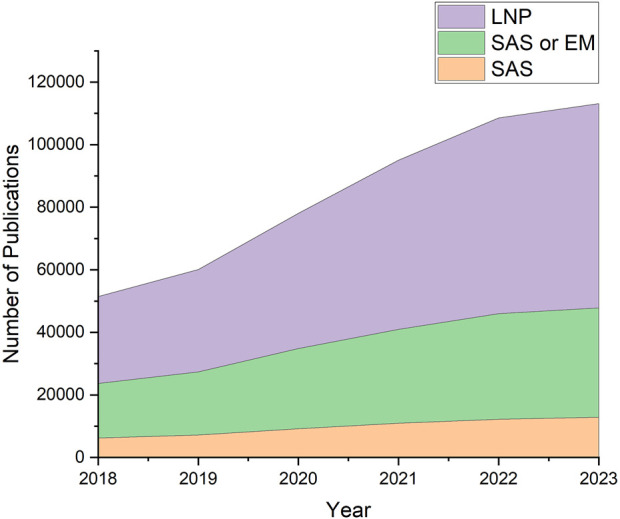
Literature search results using LNP-relevant keywords. Increase of interest in LNPs and their structures can be observed in recent years. Data was obtained from Digital Science’s Dimensions platform (Digital Science, 2018).

Lipid nanoparticles (LNPs) have been transformative for delivering oligonucleotide therapeutics. These lipid vesicles protect the nucleic acids from degradation and from triggering unwanted immune responses until they are taken up by cells (Yin et al., 2014; Dowdy et al., 2017). Safe and effective delivery of nucleic acids has been demonstrated by their application as COVID-19 vaccines (Anderson et al., 2020; Polack et al., 2020), as well as therapies to address genetic disorders and cancer (Akinc et al., 2019; Yao et al., 2020). With an established, largely successful and widespread roll-out of LNP therapeutics, there is a new interest in designing LNPs for a wide variety of applications (Tenchov et al., 2021), a process which would benefit from an understanding of the relationships between LNP composition, structure, and efficacy. Figure 1 illustrates the rise in structural investigations of LNPs, as shown by the steady increase of publications containing keywords specific to LNPs from 2018 to 2023.

LNPs form a variety of structures, ranging from vesicles to complex nanostructures that are encapsulated by a lipid layer, such as cubosomes (Barriga et al., 2018; Mohammad et al., 2022; Tan et al., 2022; Sivadasan et al., 2023), hexosomes (Abdel-Bar et al., 2020; Mohammad et al., 2022; Tan et al., 2022), and spongosomes (Chen et al., 2015; Zou et al., 2017). Detailed descriptions of the various lipid-based nanoparticles can be found in reviews by Tenchov et al., Zhai et al., and Thi et al. (Zhai et al., 2019; Tenchov et al., 2021; Thi et al., 2021). LNPs have been reported to exhibit multitudes of internal structural features, including inverse hexagonal lipid phase (Yanez Arteta et al., 2018; Aburai et al., 2018; Carrasco et al., 2021), unilamellar (Leung et al., 2012; Kulkarni et al., 2018; Nogueira et al., 2020; Patel et al., 2020; Carrasco et al., 2021), multilamellar (Uebbing et al., 2020; Fan et al., 2021), disordered core (Kulkarni et al., 2018), and combinations of such phases. In addition, phase-separate lipid-RNA cores forming ‘bleb’ structures have also been reported (Cheng et al., 2023; Thelen et al., 2024). Small variations in LNP composition can greatly affect their size, internal structure and chemistry.

The strong dependence of structure on the chemical composition is particularly challenging as LNPs are typically an assembly of many types of molecules. For example, the Pfizer/BioNTech and the Moderna COVID-19 vaccines carry nucleic acid therapeutics and are formulated from a mixture of cationic ionizable lipids, helper phospholipids, cholesterol, and polyethylene glycol-lipids (PEG-lipids). Cationic ionizable lipids facilitate the encapsulation of negatively-charged oligonucleotides and their delivery from inside the endosomes (Maier et al., 2013). Helper phospholipids and cholesterol stabilize the nanoparticle structure and aid in intracellular delivery of the cargo (Cheng and Lee, 2016). PEG-lipids modulate the surface properties of LNPs, including particle size and zeta potential, and affect their interactions with the environment, which can impact circulation time and clearance rate (Mui et al., 2013; Ryals et al., 2020; Kim et al., 2021). The choice of each lipid components (Nogueira et al., 2020; Patel et al., 2020), ratios between the different lipids (Yanez Arteta et al., 2018; Kulkani et al., 2018), ratios between lipids and nucleic acids (Kulkarni et al., 2018; Carrasco et al., 2021), as well as the type and length of oligonucleotide cargo (Aburai et al., 2020; Li et al., 2023), are all factors that could be fine-tuned for desirable outcomes.

In addition to variations in common chemical components, a host of other parameters can also dramatically affect LNP size, structure and chemistry. For example, new chemistries have been explored, such as the covalent addition of peptides to lipids that may aid in targeting LNPs to specific cells (Endsley and Ho, 2012). The physical mixing process to form LNPs can be done with various microfluidic designs or robotic handling (Chen et al., 2012; Sarode et al., 2022). pH-dependent structural changes of LNPs have also been studied to gain further insights on LNP formation and drug release (Yu et al., 2023). LNPs can change as a function of time (Ball et al., 2017), thus limiting shelf life. Storage methods may include highly concentrated solutions, freezing (−20 or −80°C) or lyophilization, and require identification of beneficial excipients. Once delivered to biological tissues, structural changes may enhance targeting and therapeutic release.

The characterization of structural features has depended on small-angle scattering (SAS) and/or cryogenic electron microscopy (cryo-EM). These techniques are complementary to each another, and provide valuable insights into the complex and dynamic LNP structures. Small-angle X-ray scattering (SAXS) and small-angle neutron scattering (SANS) provide population-wide information about the average internal structure and size of LNPs in relevant physiological conditions. SAXS has been used to characterize lipid-based nanoparticles for delivery of small-molecule drugs or macromolecular therapeutics (Angelova et al., 2011; Angelova et al., 2017; Angelova et al., 2018; Mathews et al., 2022) and the nanocomplexes they may form with biomolecules (Angelov et al., 2013; Angelov et al., 2014; Angelov et al., 2017; Yu et al., 2023). SANS can take advantage of the natural neutron contrast between lipids and nucleic acids to probe different structural regions within LNPs. As an alternative approach, SANS can exploit the difference in scattering length density (SLD) between hydrogen (^1^H) and deuterium (^2^H or D) by isotopic labeling of LNP components to selectively highlight structural regions within LNPs, thus providing detailed insights into LNP morphology (Di Cola et al., 2016; Sebastiani et al., 2021; Leite et al., 2022; Urimi et al., 2022; Yang et al., 2022; Kangarlou et al., 2023). SANS can also provide quantitative information regarding the distribution of molecules within the LNP structure, including water content in solvated regions. Cryo-EM provides direct visualization of the size and morphology of individual particles in hydrated frozen thin films (Kulkarni et al., 2018; Yanez Arteta et al., 2018; Aburai et al., 2020).

In a recent example, SANS, in combination with SAXS and cryo-EM, was used to structurally characterize a non-spherical morphology of LNPs containing self-amplifying mRNA (SAM-LNPs). SAMs are significantly larger, approximately three to four times, than conventional mRNA. SAM can generate many antigen-encoding mRNA copies and prolong expression of the antigen with lower doses than those required for conventional mRNA-LNPs. Cryo-EM showed that SAM-LNPs had two-compartments surrounded by an external shell. This non-spherical morphology was rationalized as LNPs comprising of lipid and aqueous RNA-rich phase-separated cores, both surrounded by a distearoylphosphatidylcholine (DSPC)/PEG lipid shell, also reported as ‘bleb’ LNPs (Thelen et al., 2024). Modeling of SANS data using partially deuterated LNPs indicated important structural features, including the overall size of aqueous RNA and lipid-rich compartments, water content in the aqueous RNA core, and the overall size of SAM-LNPs. Although this non-spherical morphology is not intrinsically unusual, it has been adopted by a variety of other RNA-LNPs (Kulkarni et al., 2018; Brader et al., 2021).

Compared to the vast parameter space involved in LNP formulation and the wide range of observable phases, SANS and cryo-EM have limited throughput. Furthermore, linking LNP morphologies to function requires a comprehensive understanding of LNP structure-activity relationships (SARs). Structural analyses that match or exceed the pace of producing different LNP formulations and assaying their activity will greatly enhance LNP development. Recently, HT-SAXS has been shown as a promising technique that can match the throughput of LNP formulation and provide structural information to link to cellular activity data (Hammel et al., 2023). Upon generating LNP libraries in an automated and multiplexed process, structural characterization was accomplished via HT-SAXS platform at the SIBYLS beamline (Hura et al., 2009), and efficacy data were obtained through high-throughput *in vitro* assays. Deconvolution of SAXS data using Lorentzian functions that represent different phases elucidated finer structural details that were correlated with cellular activity. For delivering short antisense oligonucleotides (ASOs), the study showed that efficacy was linked to the structural order in the core of LNPs influenced by the choice of PEG-lipids.

As the amount of data generated for LNP analyses increases, sharing the data on a platform that is easily accessible by the global community becomes critical. As demonstrated by the Protein Data Bank (PDB) (Berman et al., 2007), a comprehensive data bank with unified formats and standards empowers the field of structural biology and its related research areas. Publicly available structural data and models guide characterization of biochemical processes and inspire the development of bioinformatics and machine learning-assisted tools for analyses and predictions of biomolecular structures (Gainza et al., 2020; Baek et al., 2021; Jumper et al., 2021; Renaud et al., 2021). Without such repositories, structural data only exist as figures in the Supplementary Information of scientific articles, scattered entries across different databases, or files on a local drive that are inaccessible to the public.

The Small Angle Scattering Biological Data Bank (SASBDB) is a data archive specifically for SAS data (Kikhney et al., 2020). The primary purpose of SASBDB is to make analyses of SAS profiles available to the public and provide their computation of the underlying atomic structures of the macromolecule being studied. SASBDB is an excellent and leading resource for the SAS community to impose standards for these kinds of studies, as set out in community guidelines. Structural biologists who are not experts in the interpretation of SAS data can use the atomic models within the SASBDB when they become available, along with access to the primary data to test additional models or hypotheses. However, SASBDB contains only a few entries on lipid nanoparticles (Flayhan et al., 2018). This is, in part, because the guidelines that have been established are specific for homogeneous solutions of macromolecules and not heterogenous, mixed-density LNPs. Moreover, experiments that are likely to lead to breakthroughs in the LNP field are not singular measurements but will require integration of information from tens to thousands of measurements.

As discussed above, the large and multi-faceted parameter space of LNPs is a major challenge for optimizing formulations. If previous SAS results are to direct the optimization of a new formulation, an appropriate repository for scattering data from LNPs is indispensably necessary. Connecting an individual scattering profile from an LNP to a real space model requires comparisons to many other scattering profiles, where small changes are made and where additional data from lower throughput techniques are available. The ability to compare and contrast LNP scattering across the community would expedite the process of linking formulation to structure and efficacy. If a comprehensive data repository was available for LNPs, hundreds and thousands of scattering data generated through the high-throughput workflow could be found on a single platform to be mined and analyzed for a deeper understanding of LNP structure-activity relationship.

However, complete standardization of required metadata as done in the PDB or SASBDB may not be practical, and developing a repository that anticipates all variables is likely impossible. This challenge is not unique to LNP data, but is an emerging need across science domains. Several models have been proposed to deal with the diversity of metadata driven by the heterogeneity of scientific experiments. For example, the NWB neurophysiology data standard (Rübel et al., 2022) supports formal, user-defined extension to the data model, and the CORAL system (Novichkov et al., 2022) uses the concepts of micro-typing and contextons to support flexible extension of the data model while adhering to FAIR principles. The Linked Open Data Modeling Language (LinkML) (Moxon et al., 2021) is further an emerging standard for defining standard data schema for linked data integrated with ontologies while enabling integration with a broad range of application models, e.g., JSON-Schema, ShEx, RDF, OWL, GraphOL, and SQL DDL. Here, we propose an intermediate solution with a core metadata model is needed that is strict where possible but easy for users to extend the data model to add metadata fields not covered by the core standard. This would facilitate compliance with FAIR principles through linking of metadata to ontologies and linking of data to related datasets.

## 2 Simple Scattering

We have begun modifying a data repository we maintain called “Simple Scattering” (simplescattering.com) for the storage of LNP data. Simple Scattering is an open data repository that provides a space for SAS data from high-throughput LNP screening experiments. This data bank was designed to hold SAS data generated from various methods including size-exclusion chromatography and time-resolved experiments (Murray et al., 2023). However, it differs from other SAS repositories such as SASBDB (Kikhney et al., 2020) and BioISIS (Hura et al., 2009), which store extensively analyzed and curated SAS curves and any models derived from these data. Instead, Simple Scattering holds correlated SAS curves generated from a set of related experiments, such as buffer screens, allowing an intuitive and quick deposition of multiple, related datasets by users. Once the user decides to make the datasets public, deposited data entries are downloadable, easily searchable with keywords, and associated with unique, persistent, digital identifier codes. These data entries serve not only as storage and records of scattering data, but also as supplementary data linked to a publication for data validation.

With Simple Scattering, our goal was to create a flexible deposition system inspired by some aspects of Zenodo, while also providing sufficient standardization of data and metadata so that data is findable and accessible via application programming interfaces (APIs) and reusable for computational analyses and machine learning in adherence with FAIR principles. We do not intend to provide a direct and individualized connection of each SAS curve to real space models. We seek to design a system that provides access to the primary scattering data along with a thorough understanding of the formulation parameters including a complete description of composition. The development of Simple Scattering is also connected to an HT-SAXS synchrotron beamline (SIBYLS at the Advanced Light Source in Berkeley, CA) such that data collected at the beamline is immediately uploaded to Simple Scattering after minimal processing (Murray et al., 2023). Significant quantities of data are available with more expected as the application of SAXS to LNPs grows. The repository is open for participation by others, enabling the opportunity for a greater collection of LNP scattering data. In this broader context, we have also been actively collaborating with colleagues at the Biological Small-Angle Neutron Scattering Beamline (Bio-SANS) at Oak Ridge National Laboratory. This collaboration aims to extend the scope of Simple Scattering to include neutron scattering data, further enriching the content of the repository, and facilitating valuable insights into the structure of LNPs. A SANS dataset of LNP is currently deposited and we expect many others from the SANS and LNP communities to be deposited in the future.

Thus far, the majority of the datasets in Simple Scattering have consisted of proteins and nucleic acids. Currently, Simple Scattering holds LNP data entries from 3 publications. Hammel et al. utilized SAS to investigate the effect of PEG-lipids on structure and activity of LNPs loaded with antisense oligonucleotides (ASO-LNPs, dataset codes: XSDM4ADX, XSSOSU8C, XSK1IXUI), varying the type of PEG-lipid as well as their relative molar ratios to other lipid components (Hammel et al., 2023). O’Brien Laramy et al. performed a comparative evaluation of two different formulation methods of preparing ASO-LNPs, coaxial turbulent jet mixing *versus* microfluidic mixing (dataset code: XS0ZP76H, O’Brien Laramy et al., 2023), and Thelen et al. used a combination of different structural techniques, including cryo-EM, SAXS, and SANS to structurally characterize LNPs encapsulating self-amplifying mRNA molecules (dataset code: XSXEB4SL, Thelen et al., 2024). Numerous literature precedence exists for studying LNPs using SAS: interactions between proteins and LNPs (Flayhan et al., 2018; Sebastiani et al., 2021; Barriga et al., 2022); mixing parameters and post-formulation dilution (Hassett et al., 2021); composition ratios (Yanez Arteta et al., 2018; Barriga et al., 2022); lipid component screens (Aburai et al., 2020; Patel et al., 2020; Carrasco et al., 2021; Wilhelmy et al., 2023); and pH (Li et al., 2023). As the parameter space of LNP formulation continues to be explored, we anticipate a tremendous growth in the number of datasets collected. To reflect the increase in demand for SAS analysis of LNPs, Simple Scattering is undergoing changes to better accommodate datasets related to lipid nanoparticles.

### 2.1 Data entry system

Previous to the incorporation of LNP datasets, the Simple Scattering site was geared towards SAXS only, and in the context of biological samples. Later, we incorporated the ability to upload and share any type of SAS experiment, including SANS. Given that the site was focusing on biological samples, our users suggested additional records for typical protein and nucleotide databases and their associated IDs, such as the addition of a field for the UniProt database for proteins. Since these fields are options for their specific use case, they would simply be null for LNPs and other macromolecules or particles. Instead of simply expanding the number of fields for numerous databases and IDs to include information for LNPs, we will reconfigure both the database and the website form views to handle multiple databases within a single associated database table column, and an associated database ID column. This is in anticipation that once LNPs are introduced and flourish on the site that other communities that study different nanoparticles may also be included. For lipid nomenclature and classification scheme, Lipid Maps Structure Database (LMSD) (Sud et al., 2007) may be used, and valuable information can be provided from other databases or collections such as LIPIDAT (Caffrey M and Hogan J, 1992) through our planned APIs. In addition, we are building the database so that it is extendable to other hierarchical assemblies with similar needs for characterization.

**FIGURE 2:**
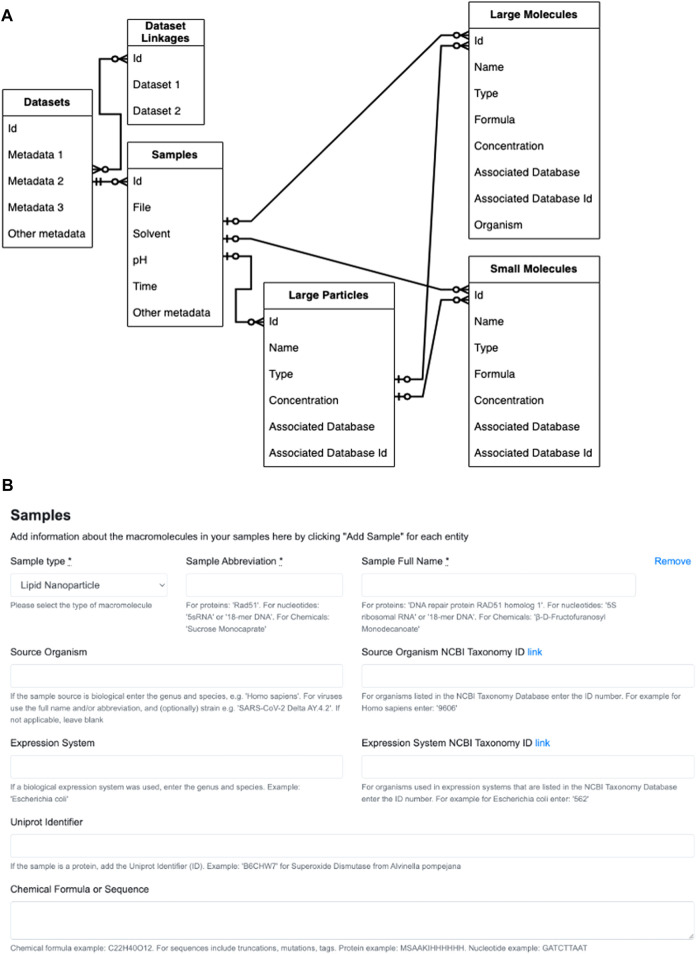
Reconfiguration of Simple Scattering database and website form views. **(A)** Entity relationship diagram for datasets, experiments or samples belonging to the dataset, and the particles and molecules that make up the sample. The table on the far left represents a single Dataset entry into Simple Scattering (for simplicity, specific metadata are not listed). The Dataset then must have one or more experiments or samples, and records that are common such as filenames, solvent and pH, will be included in this table. Each experiment must have one or more larger (nano)particles or larger molecules. One or more small molecule components (buffer) may also be included as part of the composition of each sample. Legend for arrow heads within the diagrams: 1) Double crossed: mandatory one. 2) Single-crossed with circle: optional one–note, however, it is mandatory to have one large (nano)particle or large protein. 3) Circle chickenfoot: optional many. **(B)** The original sample information form geared towards proteins and oligonucleotides.

As described above, effectively recording important and often non-standard metadata for LNP samples is a challenge. Typically, LNP formulations are described using 1) percentage ratios of lipid components that were mixed together and 2) the final concentration of the LNPs in the buffer solution. In case LNP samples were also studied using SANS, the deuteration levels of lipids or nucleic acids also need to be recorded in the metadata. While the concentrations of each component may easily be converted from being relative to a larger mass to that of the solvent, the conversions may introduce floating point errors when going back and forth between the database and the website views. Therefore, we are reengineering the site’s database and rewriting the code for the associated models, controllers and views such that the following will occur (Figure 2A): 1) General Dataset metadata will be stored in a Dataset table. 2) Each Dataset must contain at least one experiment or sample. 3) The composition of the sample under study or the subject of the experiment must contain at least one larger particle (LNP or other nanoparticles) made up of large molecules, or at least one large molecule (protein, DNA, etc.). For example, a lipid nanoparticle sample (“large particle”) can be blank (no “large molecule”) or contain DNA as its active ingredient (“large molecule”). A sample consisting of only proteins and/or nucleic acids will not contain a “large particle.” 4) Each sample or experiment must include at least one small molecule if there is any type of buffer component; otherwise, small molecules may be omitted if the large particle or large molecule is in just a single solvent such as water. The smaller molecules are generally buffer components that will also be subjected to SAS for the purpose of background subtraction. For instance, if deuterium oxide (D_2_O) was used in a SANS experiment, it can be recorded under small molecule metadata. Other typical small molecules may include excipients such as sucrose and glycerol.

Currently, sample information is entered manually in pre-designated blanks or reported in lists within a “File description” text box (Figure 2B). To make the ability to fill in the form to describe many samples quickly, we will allow an option to upload comma-separated values (csv) in the form of a file. To make generating csv files easier, we will implement several web-based templates, downloadable scripts that can build csv files, and possibly custom conversion programs for regular users. These tools for generating csv files will allow users to provide minimal information necessary to replicate their formulation process, such as lipid components, deuteration levels, and their relative ratios, and enable facile data mining for any future machine learning analysis.

### 2.2 Linking related data entries

Another useful feature to be added is the ability to link related data entries. Since data collection for HT-SAXS occurs much more rapidly than SANS, HT-SAXS could be used for screening and SANS experiments could be run to provide a deeper insight into the internal structures of a few representative samples. Therefore, we will also join these together within the website’s database along with links within the website views that are determined by the Researcher that is uploading their data to share. By linking the SAXS and SANS data collected from identical samples, the relationship between the two datasets can be made clear to the public.

While in-depth analyses of SAS curves will occur prior to deposition and outside of the repository, users will also benefit from being able to visualize the scattering profiles directly from Simple Scattering. Additionally, since HT-SAXS data collection from a 96-well plate containing LNP libraries takes only 1.5 h (Hura et al., 2009), the number of scattering data will escalate quickly and make manual comparisons between data difficult. Therefore, implementation of SAXS Similarity (Hura et al., 2013) to automatically generate a structural comparison map will provide a useful, global overview of data and instantly identify clusters of similar scattering profiles. With these developments to come, Simple Scattering will hopefully become a resource that is more useful and easier to utilize for LNP research communities.

## 3 Discussion

Due to the complexity of lipid nanoparticles governed by many factors including different lipid components and nucleic acids, making direct associations between the formation of different structural phases and lipid compositions is difficult. This challenge is exacerbated by the limited throughput of techniques and resources to study them. However, with automation and robotics continuing to increase the throughput of experiments, the scope of experimental parameters that can be tested has expanded dramatically. As demonstrated by Hammel et al., the field of LNP development can leverage the high-throughput capabilities in formulation, structural analyses, and cellular activity assays to explore the vast chemical space and factors involved in LNP drug design and optimization (Hammel et al., 2023). Rapid data collection via HT-SAXS, combined with complementary methods such as SANS, may expedite the creation of far more effective vaccines when necessary.

During the high-throughput LNP workflow, large volumes of data will be generated, and we have introduced Simple Scattering as a data bank to store scattering data in an organized and accessible manner. While limitations do exist, Simple Scattering provides a starting point for depositing thousands of scattering profiles generated using HT-SAXS and SANS, and with the continual growth of interest in LNPs, we envision that the database will grow rapidly. As the current shortcomings are addressed by the upcoming changes, more input and suggestions from the broader community of scientists working with SAS and/or LNPs would be greatly appreciated to develop this repository into a collaborative and useful resource for all. As groups around the world screen for conditions to achieve optimal LNP performance and deposit the resulting data on Simple Scattering, this data bank will prevent unnecessary and unintentional replications of experiments and provide training datasets for future analyses assisted by computational resources. Aided by various machine learning algorithms, key conditions for achieving optimal activity may be identified, and predictions of LNP structure may be made based on specific combination of lipid components and relative molar ratios. Ultimately, we hope to develop Simple Scattering into a collaborative and collective knowledge base to improve our understanding of LNPs and facilitate the development of future LNP therapeutics with optimal performance.

## Data Availability

The original contributions presented in the study are included in the article/Supplementary material, further inquiries can be directed to the corresponding author.
